# Anti-Arthritic and Immunomodulatory Potential of Methanolic, n-Hexane, and Ethyl Acetate Fractions of Bark of *Acacia modesta* on Complete Freund’s Adjuvant-Induced Arthritis in Rats

**DOI:** 10.3390/pharmaceutics15092228

**Published:** 2023-08-29

**Authors:** Kiran Mashaal, Arham Shabbir, Mahtab Ahmad Khan, Huma Hameed, Muhammad Shahzad, Ali Irfan, Gamal A. Shazly, Aisha Mobashar, Tasleem Akhtar, Zaib Ali Shaheryar, Yousef A. Bin Jardan

**Affiliations:** 1 Department of Pharmacology, Faculty of Pharmacy, The University of Lahore, Lahore 54000, Pakistan; 2 Department of Pharmacology, Institute of Pharmacy, Faculty of Pharmaceutical and Allied Health Sciences, Lahore College for Women University, Jail Road, Lahore 54000, Pakistan; 3 Faculty of Pharmaceutical Sciences, University of Central Punjab (UCP), Lahore 54000, Pakistan; 4 Department of Pharmacology, University of Health Sciences, Lahore 54000, Pakistan; 5 Department of Chemistry, Government College University Faisalabad, Faisalabad 38000, Pakistan; raialiirfan@gmail.com; 6 Department of Pharmaceutics, College of Pharmacy, King Saud University, Riyadh 11451, Saudi Arabia; 7 Institute of Experimental and Clinical Pharmacology and Toxicology, University of Lübeck, 23562 Lübeck, Germany

**Keywords:** inflammatory markers, cytokines, rheumatoid arthritis, immunomodulation, methanolic extract

## Abstract

Rheumatoid arthritis is an autoimmune disorder and topic of interest for researchers due to its increasing frequency and limited treatment. *Acacia modesta* Wall is known to treat rheumatic disorders in the traditional system of medicinal plants. Traditional medicines are still required for the treatment of this disease due to the large number of side-effects caused by commercial medicines. In the current study, the antiarthritic potential of methanolic extract (AM-metha), n-hexane (AM-hexa) fraction, and ethyl acetate (AM-etha) fraction of the bark of *A. modesta* against a complete Freund’s adjuvant rat model was evaluated. Evaluation using a digital plethysmometer, macroscopic evaluation, and histopathological evaluation were conducted to determine the paw volume and arthritic scoring. ELISA was performed to assess the PGE2 levels. RT-PCR was used to evaluate the expression levels of MMP2, MMP3, MMP9, NF-κB, IL6, IL1β, TNFα, and VEGF. Biochemical and hematological analyses were also conducted. GC/MS was also carried out to analyze the presence of medicinal compounds. The data revealed a marked reduction in the paw volume, arthritic scoring, and histopathological parameters, indicating the anti-arthritic potential of the plant. Treatment with plant extracts and fractions markedly down-regulated MMP2, MMP3, MMP9, NF-κB, IL6, IL1β, TNFα, and VEGF levels. Similarly, PGE2 levels were also found to be ameliorated in the treatment groups, indicating the immunomodulatory property of plant bark. Plant treatment nearly normalized hematological parameters such as counts of WBCs, RBCs, and platelets, along with Hb content, thereby validating the anti-arthritic activity. GC/MS analysis disclosed the presence of strong anti-inflammatory compounds such as lupeol, oleic acid, and squalene. The study showed that *A. modesta* possesses anti-arthritic and immunomodulatory potential linked to significant down-regulation of pro-inflammatory and inflammatory biomarkers.

## 1. Introduction

Inflammation is a natural reaction in the body and occurs due to many endogenous and exogenous factors [1]. Swelling, pain, heat, redness, and loss of function are the signs of inflammation [2]. Rheumatoid arthritis (RA) is a chronic disorder that is responsible for the inflammation of synovial joints and hypertrophy [3]. RA affects about 0.5–1% of the population of the world and it is three times more likely in women than in men [4]. RA severely affects the ankle joints, causing pannus development, eroded bones and cartilage, and inflamed synovial membrane, and causes many changes in the morphology of joints [5]. Despite the fact that inflammation is known to be a biological host defense response, chronic inflammatory responses are considered to be a major cause of RA [2].

The etiology of this disease is still not fully understood, but many immune pathways, including those of immune and non-immune cells, contribute to the release of various proinflammatory cytokines, chemokines, proteases (MMPs), and other matrix-lysing enzymes, resulting in cartilage and bone damage [6]. Higher levels of pro-inflammatory cytokines (e.g., TNFα, IL1, and IL6) caused by an imbalance between pro- and anti-inflammatory cytokines leads to the pathogenesis of RA [7]. TNF-α is involved in the damage of bone and cartilage via the enhancement of other pro-inflammatory cytokines and osteoclast formation [8]. IL6 is another immunomodulator that is actively involved in the pathogenesis of RA [9]. The activation of IL1β in rheumatoid synovium up-regulates the matrix metalloproteinase (MMP) levels, which mediates synovial inflammation, leading to damage of bones and joints [10]. The MMPs are considered to be responsible for the degradation of cartilage in the inflamed joints [11]. PGE2 is another inflammatory mediator that participates in the degradation of bone and cartilage via activation of MMPs [12]. As a mediator of angiogenesis and inflammation, vascular endothelial growth factor (VEGF) also contributes to the pathogenesis of disease [13]. NF-κB is not only a recognized regulator of inflammation in RA, but is also involved in broader aspects of RA pathology such as abnormal apoptosis, producing a Th1 response and stimulation of osteoclast activity [14,15]. Considering the significant role of these mediators in the pathogenesis of RA, the effects of a plant extract and its fractions on expression levels of these mediators were evaluated in the current study. 

Many pharmacological agents, such as conventional synthetic disease-modifying antirheumatic drugs (DMARDs), targeted synthetic DMARDs, and biologic DMARDs, are given to treat RA in order to maintain joint activity. Nonsteroidal anti-inflammatory drugs (NSAIDs) and glucocorticoids (GCs) are used as an adjunct and as a symptomatic treatment for inflammation [16]. However, severe adverse effects of these treatments are reported due to their high dose and long-lasting usage [17]. Plants and their constituents are receiving recommendations due to their maximum pharmacological efficacy, minimum adverse reactions, and low costs, especially for inflammatory and immunomodulatory disorders [18]. Globally, herbal medicines are now gaining importance as traditional systems of medicine. Constituents isolated from them have been reported to be active constituents in drug discovery [19]. 

*Acacia modesta* Wall is an average sized tree belonging to the family Mimosaceae, and is widely found in rocky ground in India and Pakistan. The tree is locally called Phulai and Palosa [20]. *Acacia modesta* bark has been traditionally used for the treatment of rheumatic disorders [21,22]. Locally, *A. modesta* is used for the relief of various bone-, muscle-, and stomach-related disorders [23]. Research studies have shown that *A. modesta* possesses analgesic, anti-inflammatory, antiplatelet [20], antioxidant [24], anti-hyperglycemic [25], antidiarrheal, antisecretory, antispasmodic [26], antimicrobial [27], antidepressant, and anticoagulant [28] activities. 

The current study aimed to determine the antiarthritic potential of AM-metha (methanolic) extract, AM-hexa (n-hexane fraction), and AM-etha (ethyl acetate fraction) of the bark of *A. modesta* Wall against a complete Freund’s adjuvant (CFA)-induced rat model of arthritis.

## 2. Materials and Method

### 2.1. Extract Formation and Fractionation

The bark was taken from the Bheen village, Chakwal district, Punjab province, and was identified by Dr-Zaheer-ud-din Khan, Government College University, Lahore (GC.Herb.Bot.3542). The fine powder (1 kg) was poured into methanol (3 L) in a dark container. After 1 week, the material was filtered through a filter sheet (Whatman number 1). Then, the filtrate was concentrated under reduced pressure using a rotary evaporator (Lab tech EV3111plus) [29]. The percentage yield of methanol extract of *A. modesta* was calculated to be 10.6%.

The fractionation was undertaken by dissolving methanol extract in 500 mL of distilled water and then the mixture was mixed with 500 mL of n-hexane in a separating funnel. Then, the n-hexane fraction was collected and concentrated under reduced pressure using a rotary evaporator. The n-hexane fraction yield was calculated to be 4%. Later, the same procedure was repeated to obtain the ethyl acetate fraction. The ethyl acetate fraction yield was calculated to be 4.3% [30]. 

### 2.2. Animal Housing

Sprague Dawley rats (150–250 g and 6–8 weeks old) of both genders were used for this study. Standard animal diet, humidity (40–60%), 12 h dark/light cycle, and temperature (24–26 °C) were provided to the rats in the animal house of the Faculty of Pharmacy, The University of Lahore. The study was conducted according to national, international, and institutional ethics guidelines regarding animal experiments under the approval number IREC-2019-91, issued by the Institutional Research Ethics Committee, The University of Lahore [31]. 

### 2.3. Treatment Design and Induction of Arthritis

For this study, 6 groups were created and each group had 6 rats. CFA at a dose of 0.15 mL (Santa Cruz biotechnology) was injected at day 0 to develop arthritis in the sub-plantar area of the left hind paw in all groups except the normal control. The dosing was started on the 8th day and ended on the 22nd day. After 24 h on day 23, all rats were sacrificed to begin the study [29]. All treatment groups are shown in Table 1.

### 2.4. Arthritic Score and Paw Volume

A macroscopic scoring method was used to measure the visible signs, such as inflammation, redness, and swelling, in the injected paw at days 8, 13, 18, and 23 of arthritic induction. Score 0 indicated normal, 1 indicated minor, 2 indicated mild, 3 indicated moderate, and 4 indicated severe signs [34]. The paw volume was measured using a water displacement digital plethysmometer (LE 7500, Panlab, Barcelona, Spain) at days 8, 13, 18, and 23 after arthritic induction. The non-injected paw was taken as the negative control. The volume of the non-injected paw was subtracted from the volumes of the injected paw to estimate the increase in paw volume. In addition, % inhibition was also calculated [35]. 

### 2.5. Ankle Joint Histopathology

After euthanasia, the ankle joints were separated, fixed, and decalcified. The paraffin blocks were sliced at a thickness of 5 μm and then stained with hematoxylin and eosin (H&E). The histopathological markers of arthritis were evaluated by a blinded histopathologist using 0–4 semi-quantification criteria [36].

### 2.6. Biochemical and Hematological Biomarkers

Alkaline phosphate (ALP), alanine aminotransferase (ALT), aspartate aminotransferase (AST), creatinine, total bilirubin, and urea levels were measured in serum samples as part of biochemical analysis using a chemistry analyzer and the kit protocol. Platelet, white blood cell (WBC), red blood cell (RBC), and hemoglobin (Hb) levels were measured in blood samples using an automatic hematology analyzer [37]. 

### 2.7. PGE2 Protein Levels

The sandwich ELISA method as per the kit protocol (Bioassay technology laboratory, Shanghai, China) was used for measuring PGE2 levels in serum samples. Optical density (OD) was observed at 450 nm [17].

### 2.8. Determination of MMP2, MMP3, MMP9, NF-κB, IL6, IL1β, TNFα, and VEGF Expression Levels

Total RNA from the blood samples was extracted using the TRIzol method. The samples were processed with chloroform, isopropyl alcohol, and 75% ethanol for RNA extraction. After extraction, the RNA samples were processed to determine their quantity and purity using a nanodrop spectrophotometer [38]. Each sample containing 1000 ng RNA was reverse transcribed according to the kit manufacturer’s protocol (Thermo Scientific, Waltham, MA, USA) to synthesize cDNA [39]. Amplification of cDNA (1µL) was performed by mixing it with forward primer (1 µL), reverse primer (1 µL), nuclease-free water (2 µL), and PCR Master Mix (5 µL) (Thermo Scientific). This PCR mixture was placed in a thermal cycler, which was programed for 39 cycles of denaturation, annealing, and extension. Ensemble Genome Browser online was used to design gene-specific primers of MMP2, MMP3, MMP9, NF-κB, and IL-1β. Primer sequences of IL-6, TNFα, and VEGF were chosen from previous articles, as shown in Table 2. GAPDH served as the housekeeping gene. The obtained PCR product was visualized using gel electrophoresis and a gel documentation imaging system, while ImageJ software 1.47t was used for densitometry [40].

### 2.9. GC/MS Analysis

GC/MS (GCMS-5975c) analysis was performed to determine the presence of constituents in bark extract and fractions. The analysis was performed using capillary column # HP-5MS (30 m × 250 μm × 0.25 μm) and helium with the flow rate of 1 mL/min was used as an inert gas. The oven temperature was initially programmed at 60 °C for 0 min; the temperature was then decreased at 5 °C/min and increased to 80 °C for 2 min, and then decreased at 10 °C/min and increased to 310 °C for 4 min. The total run time of the solutions was 33 min. The relative voltage for MS was 47 and 200 °C was programmed for the quadrupole analyzer. The mass-to-charge range was set at 30 to 700. The GC/MS analysis was performed using the method published in a previous article with slight changes [35].

### 2.10. Statistical Analysis

GraphPad Prism 5 was used for statistical analysis. One-way analysis of variance and Tukey’s post hoc test were applied for analysis and comparison of groups. All data are presented as the mean ± standard deviation. Significant differences were considered at ^a^ *p* > 0.001, ^b^ *p* > 0.01, and ^c^ *p* > 0.05 when test groups were compared with the disease group.

## 3. Results

### 3.1. A. modesta Attenuated Arthritic Score and Paw Volume 

Arthritic score was increased in all groups except the normal control group at day 8. In the normal control group, disease was not developed; because the readings of this group were taken as zero, no bar chart is shown. At days 13, 18, and 23, arthritic score was gradually increased in the arthritic control group, while AM-metha, AM-hexa, AM-etha, and piroxicam treatment groups had a lower arthritic score at different day intervals. At the end of the treatment, all treated groups had a remarkably reduced score when compared with the arthritic control group.

**Figure 1:**
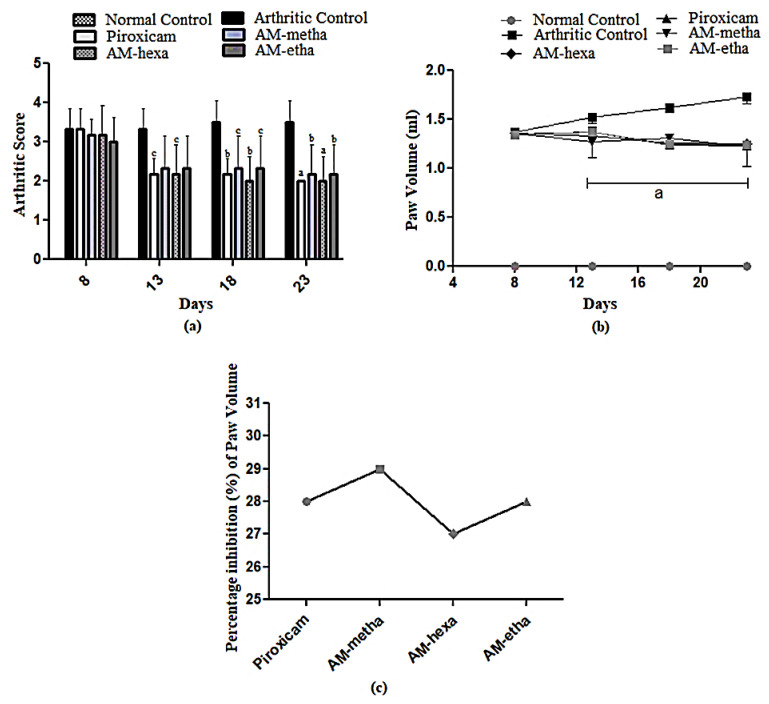
Arthritic index and paw volume. (**a**) ^c^ *p* < 0.05, ^b^ *p* < 0.01, and ^a^ *p* < 0.001, and (**b**) ^a^ *p* < 0.001 when other group values were compared with arthritic control; (**c**) % inhibition when compared with the arthritic control. The highest percentage inhibition was shown by the AM-metha group.

AM-metha, AM-hexa, AM-etha, and piroxicam significantly reduced the paw volume when compared with the arthritic control group. Percentage inhibition of paw volume was also calculated using a previously reported formula. AM-metha (29.47%), AM-hexa (27.74%), AM-etha (28.32%), and piroxicam (28.90%) inhibited paw volume in comparison with the arthritic control group, as shown in Figure 1.

### 3.2. A. modesta Ameliorated Histopathological Parameters of Arthritis

**Figure 2:**
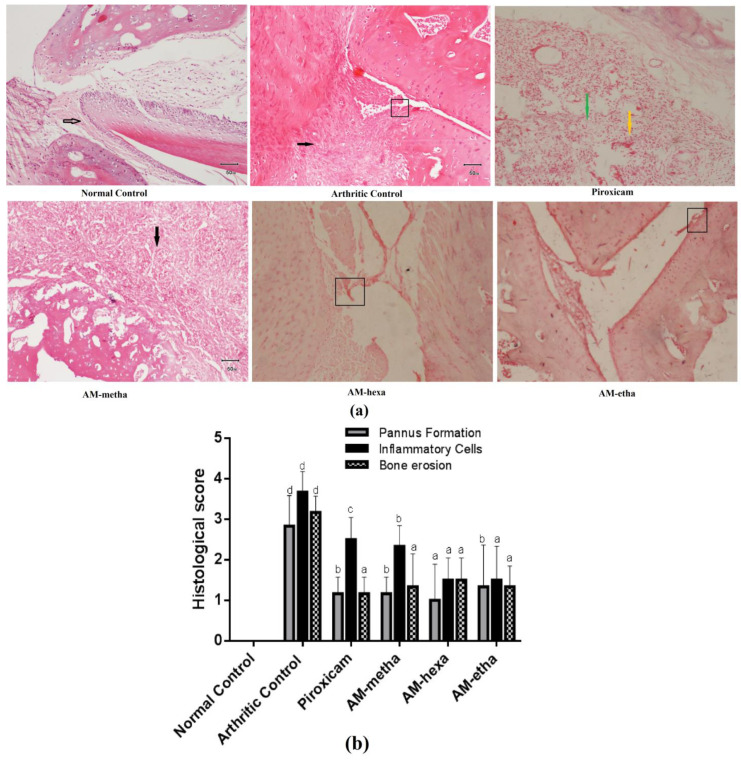
(**a**) Photomicrograph (100×) of histopathological findings (H&E stained). (**b**) Values are shown as mean ± SD. When treatment group’s values were compared with the arthritic control, then ^c^ *p* < 0.05, ^b^ *p* < 0.01 and ^a^ *p* < 0.001 were considered. ^d^ *p* < 0.001 when arthritic control was compared with normal control.

AM-metha extract, AM-hexa fraction, AM-etha fraction, and piroxicam displayed reduction in erosion of bones, inflammatory cell infiltration, and pannus formation in comparison with the arthritic control. The normal control showed normal bone and a normal synovial layer (black arrow); the arthritic control showed marked pannus formation (black arrow) and eroded cartilage (rectangular); piroxicam showed fibrosis formation (green arrow) and moderate infiltration of inflammatory cells (yellow arrow); AM-metha displayed moderate pannus formation (black arrow); AM-hexa showed eroded bone at the level of the joint (rectangular); and AM-etha showed mild erosion of joint cartilage; see Figure 2a,b.

### 3.3. A. modesta Significantly Reduced PGE2 Levels

**Figure 3:**
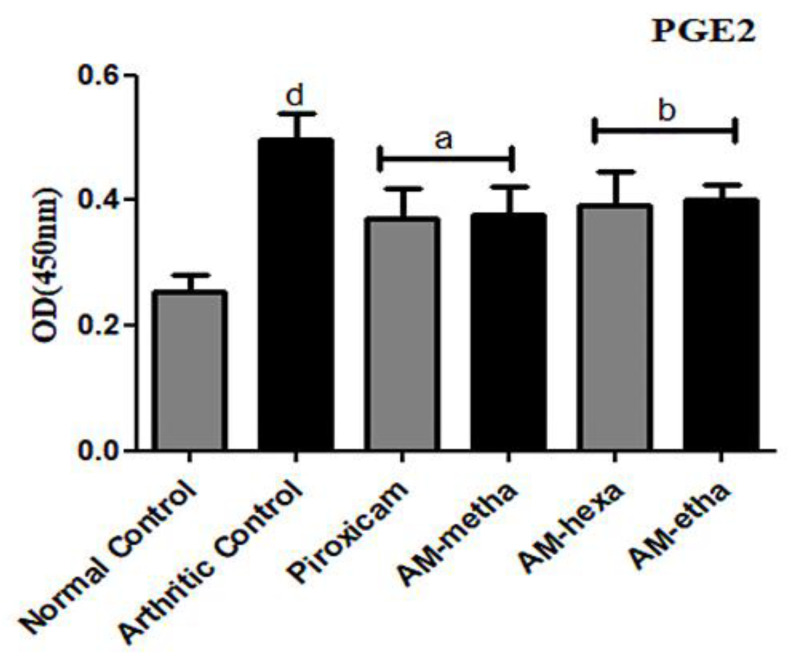
PGE2 protein level. AM-meth, AM-hexa, and AM-etha decreased the levels of PGE2 as compared to the arthritic control group. Here, ^a^ *p* < 0.001 and ^b^ *p* < 0.01 are compared to the arthritic group, whereas ^d^ *p* < 0.001 shows the comparison between the normal control and arthritic groups.

Levels of PGE2 were found to be significantly raised in the arthritic control in comparison with the normal control. The treatment with extract of AM-metha and its fractions, such as AM-hexa and AM-etha, prominently reduced the PGE2 levels when compared with the arthritic control. Piroxicam also significantly decreased the PGE2 levels as compared to the arthritic control, as shown in Figure 3. 

### 3.4. A. modesta Significantly Reduced Matrix Metalloproteinase, Pro-Inflammatory Cytokine, Transcription Factor, and Growth Factor Expression Levels

**Figure 4:**
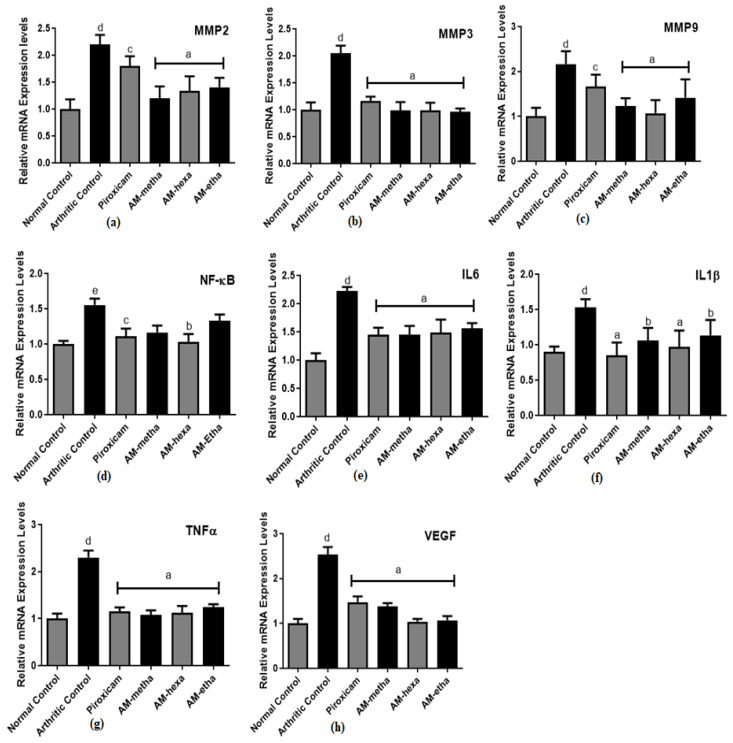
AM-metha and AM-hexa prominently reduced the expression levels of (**a**) MMP2, (**b**) MMP3, (**c**) MMP9, (**d**) NF-κB, (**e**) IL6, (**f**) IL1β, (**g**) TNFα, and (**h**) VEGF, while AM-metha and AM-etha showed a non-significant reduction in NF-κB expression levels as compared to the arthritic control group. Here, ^a^ *p* < 0.001, ^b^ *p* < 0.01, and ^c^ *p* < 0.05 are compared to the arthritic group, whereas ^d^ *p* < 0.001 and ^e^ *p* < 0.01 shows the comparison between the normal control and arthritic groups.

Expression levels of matrix metalloproteinases, such as MMP2, MMP3, and MMP9, were increased in all groups after CFA induction, but after treatment with AM-metha, AM-hexa, and AM-etha, their levels were significantly reduced when compared with the arthritic control. Expression levels of pro-inflammatory cytokines, such as IL6, IL1β, and TNFα, were also found to be higher in the arthritic control as compared with the normal control. Treatment with AM-metha, AM-hexa, and AM-etha reduced their levels significantly when compared with the arthritic control. The transcription factor NF-κB and the growth factor VEGF were significantly increased in arthritic control rats as compared to normal control rats. All treatment groups displayed a significant reduction in VEGF expression levels, while AM-metha and AM-etha showed a non-significant reduction in NF-κB expression levels as compared to the arthritic control group. Piroxicam also showed comparable results (Figure 4).

### 3.5. A. modesta Improved Hematological Markers and Did Not Display Harm to Liver and Kidneys

**Figure 5:**
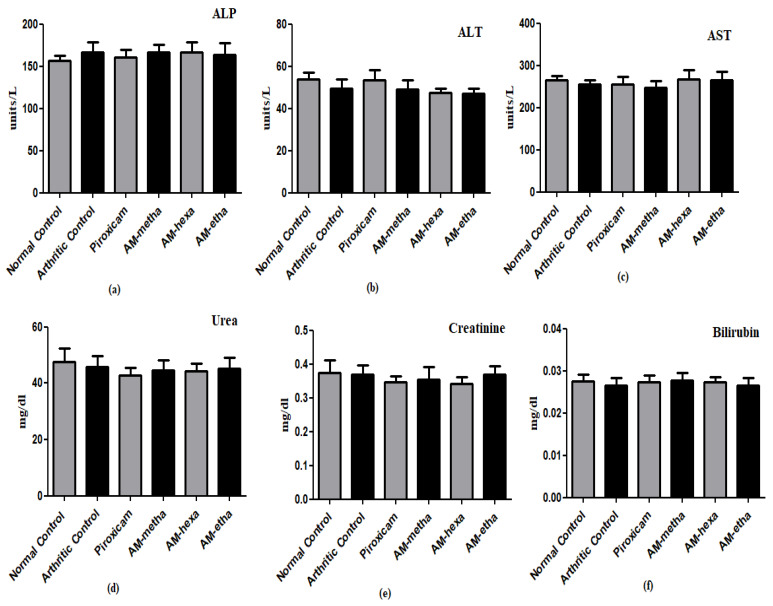
Biochemical markers. In the estimation of (**a**) ALP, (**b**) ALT, (**c**) AST, (**d**) urea, (**e**) creatinine, and (**f**) total bilirubin levels, a non-significant difference was found in all groups when compared with each other.

The arthritic control group showed reduced hemoglobin content and RBC counts as compared to the normal control. Treatment with AM-metha, AM-hexa, AM-etha, and piroxicam showed elevated Hb content and RBC counts. A significant elevation in platelet and WBC counts was observed in arthritic control rats as compared to normal control rats, respectively. AM-metha, AM-hexa, and AM-etha groups displayed a reduction in the levels. The attenuation was also seen in piroxicam-treated rats (Table 3). The levels of ALP, total bilirubin, creatinine, urea, AST, and ALT were also evaluated in the current study and a statistically non-significant difference was detected among all groups when they were compared with each other (Figure 5).

### 3.6. GC/MS Analysis of Extract and Fractions of A. modesta

GC/MS analysis of the methanolic extract, n-hexane fraction, and ethyl acetate fraction of *A. modesta* was conducted to determine the presence of anti-inflammatory compounds. The bioactive compounds, with their retention time, total percentage, molecular formula, and molecular weight, are shown in Table 4 for methanolic extract, Table 5 for n-hexane fraction, and Table 6 for ethyl acetate fraction.

## 4. Discussion

Rheumatoid arthritis (RA) is an inflammatory autoimmune disorder subsequently resulting in the formation of pannus, infiltration of inflammatory cells, and synovial hyperplasia, followed by eroded bone and cartilage [37]. In this disease, macrophage and fibroblast-like synoviocytes are the main contributory factors in pannus formation and facilitate bone and joint damage during joint inflammation [6]. The CFA rat model is the model of choice and is commonly used for the evaluation of chronic inflammatory disease due to its similarities with human RA. This model is known to trigger synovial hyperplasia, inflammation, and damage to bone and cartilage by up-regulating the pro-inflammatory cytokines and infiltrating inflammatory cells [5]. A marked inflammation was seen in the hind paw of rats injected with CFA. The vascular exudative phenomenon transports the immune cells to the site of the injected area in the acute phase of inflammation. Then, production of pro-inflammatory cytokines facilitates the formation of pannus, synovial hyperplasia, and damage to bone and cartilage in the chronic phase of inflammation [37]. In the current study, a reduction in paw volume, arthritic score, and histopathological findings was found in plant-treated groups, which showed the anti-arthritic potential of *A. modesta*.

The pro-inflammatory cytokines, such as TNFα, IL6, and IL1β, play a dominant part in the pathogenesis of RA [36,43]. Cytokine-mediated inflammation is a hallmark of autoimmune diseases. Cytokines such as IL1, 1L6, and TNFα are released from synovial tissues into the systemic circulation and participate in the degradation and inflammation of joints. The levels of 1L6, IL1β, and TNFα are found to be higher in arthritic patients [3,44]. Cytokines work in many areas of inflammatory processes associated with inflammation regulation, autoimmune events, inflammation of synovial membranes, and damage of articular joints [6]. In RA, the activation and differentiation of osteoclast are facilitated by the increased level of pro-inflammatory cytokines. IL1β takes part in the development of osteoclasts by stimulating the RANKL (receptor activator of NF-kappa B ligand), which possesses osteoclastogenic properties. The activation of IL1β in the synovium tissue causes overexpression of MMPs, leading to damage of bone and joints [10]. TNFα also up-regulates the levels of MMPs and PGE2 via fibroblasts and macrophages, leading to synovitis, bone resorption, and cartilage erosion. It also enhances the levels of another pro-inflammatory cytokine, i.e., IL6 [5], which is considered to be a multifunctional cytokine and participates in the activation of osteoclastogenesis, the inflammatory process, synovitis, and the up-regulation of MMPs, leading to bone and cartilage destruction in the affected joints [45]. Attenuation in the gene expression levels of TNFα, IL6, and IL-1β was found in plant-bark-treated groups as compared to the disease group, showing the immunomodulatory property of *A. modesta*.

MMPs are inflammatory enzymes that are involved in the damage of joints by degrading the components of the extracellular matrix [46]. MMPs such as MMP2, MMP3, and MMP9 are up-regulated in arthritis, leading to damage to bones and cartilage of joints via activation of IL1β, TNFα, and many other cytokines. These cytokines regulate the monitoring of MMPs, mainly through the mitogen-activated protein kinase (MAPK) signal transduction pathway [47]. VEGF, as an inflammatory and angiogenetic mediator, causes the activation of pro-inflammatory cytokines and the development of new vessels by stimulating the immune and endothelial cells. This activation then leads to an inflammatory and angiogenetic event in RA [13]. PGE2 is the key prostaglandin among the various prostaglandins, and is activated by synovial fibroblasts, chondrocytes, and pro-inflammatory cytokines such as IL1β and TNFα [48]. PGE2 is abundantly found in RA patients and acts as an inflammatory and pain marker in RA. It is known to damage bone and cartilage by activating metalloprotease enzymes [12]. NF-ĸB is a transcription factor that has a major involvement in the pathogenesis of rheumatoid arthritis [49]. The activation of NF-κB plays a significant role in the stimulation of pro-inflammatory cytokines and MMPs, and in the inhibition of apoptosis, leading to development of disease [48]. The levels of PGE2, MMP2, MMP3, MMP9, VEGF, and NF-ĸB were down-regulated in extract- and fraction-treated groups when compared with the disease group, validating the immunomodulatory and anti-inflammatory properties of *A. modesta*, as indicated by the down-regulation of pro-inflammatory cytokines.

The biochemical levels in serum, such as those of ALP, AST, ALT, total bilirubin, urea, and creatinine, were evaluated to determine the safety of plant bark in the liver and kidney [29]. The higher levels of liver enzymes such as ALP, AST, and ALT in the systemic circulation is an indication of hepatic damage [34]. Apart from this, ALP also acts as a biomarker of bone damage and its levels become elevated in RA [50]. Elevated levels of total bilirubin act as a prognostic indicator for acute to chronic liver disease [51]. The higher levels of creatinine and urea in the systemic circulation are a sign of renal damage because they act as a biomarker of renal disease. In the normal state, these compounds are filtered out from the blood through the kidneys, whereas in a pathological state, their levels become elevated in the circulation due to the inability of the kidneys to excrete them properly [29]. Our study found non-significant differences in the levels of ALP, ALT, AST, urea, creatinine, and total bilirubin when compared with each other, which suggests that no harm to kidney or liver was caused by plant bark treatment. The changes in the levels of hematological parameters in CFA-induced rats are linked to the severity of RA. Anemia is common in rheumatoid arthritis, characterized by low levels of RBCs and Hb content [31]. The anemic condition occurs due to the abnormality in the storage of iron in the synovial tissue and reticuloendothelial system. In addition, the inability of bone marrow to generate adequate cells is also a causative factor in the attenuation of RBCs and Hb content. Activation of the immune system against antigen attack leads to high levels of WBCs and platelets in RA [32]. Elevation of WBC count is associated with the activation of the immune system against destructive pathogens, ultimately leading to activation of inflammatory modulators [36]. Platelets play a significant role in inflammation and immunomodulation. Microparticles released from platelets interact with WBCs and produce systemic and joint inflammation in RA [52]. Treatment with plant bark increased the levels of RBCs and Hb content and decreased the levels of WBCs and platelets when compared with the arthritic control, thus the validating anti-arthritic potential of *A. modesta*.

Although not perfect, chromatography is a widely used method for the identification of constituents in extracts and fractions. The GC-MS analysis of *Acacia modesta* Wall revealed the presence of previously reported anti-inflammatory and antioxidant phytochemicals, which might be attributed to its antiarthritic property. Lupeol is known to be a powerful anti-inflammatory and antioxidant compound. It reduces the progression of arthritis through down-regulation of the P13K/Akt signaling pathway [53,54,55]. Oleic acid possess anti-inflammatory activity [56]. It produces its effect by targeting proinflammatory cytokines TNF-α and IL-1β [57]. γ-Sitosterol has both anti-inflammatory and antioxidant effects [58,59]. Squalene is reported to be an anti-inflammatory constituent [60]. Hexadecanoic acid, methyl ester, and 5-Hydroxymethyl furfural produce antioxidant effects [61,62]. All these phytochemicals and others found in this study may be credited with the anti-inflammatory activity, which resulted in amelioration of RA.

## 5. Conclusions

Treatment with *Acacia modesta* Wall. bark ameliorated rheumatoid arthritis. The data indicate that treatment with *A. modesta* reduced the paw volume, arthritic score, pannus formation, cartilage and bone erosion, and infiltration of inflammatory cells, thus showing the anti-arthritic potential of the plant. The anti-arthritic potential may be ascribed to down-regulation of MMP2, MMP3, MMP9, IL1β, TNFα, IL6, NF-κB, PGE2, and VEGF levels. Anti-inflammatory constituents such as lupeol, oleic acid, squalene, and various others found in the extract and fractions of plant bark might be credited with the anti-inflammatory activity, resulting in suppression of rheumatoid arthritis. Further studies are required for standardization of the plant extract before its consideration as a viable treatment option alongside conventional medicines.

## Figures and Tables

**Table 1:** Treatment design.

Treatment Groups	Dose	Route of Administration	References
Normal Control	Normal Saline	Orally	[29]
Arthritic Control	i. CFA ii. Normal saline	i. Injected ii. Orally	
Piroxicam	10 mg/kg b.w to arthritic rats	Intraperitonially	[32]
AM-metha	500 mg/kg b.w to arthritic rats	Orally	[33]
AM-hexa	500 mg/kg b.w to arthritic rats	Orally	
AM-etha	500 mg/kg b.w to arthritic rats	Orally	

**Table 2:** Primer list and sequences.

Markers	Forward/Reverse Primers	Annealing Temp (°C)	Product Size	References
IL6	5′-GTCAACTCCATCTGCCCTTCAG-3′ 5′-GGCAGTGGCTGTCAACAACAT-3′	59	270	[41]
MMP3	5′-CCTTTTGATGGGCCTGGAAT-3′ 5′-GTGACATCATCTTGTCCATCG-3′	54	107	ENSRNOG00000032626
IL1β	5′-CCTGCTAGTGTGGATGTTC-3′ 5′-GAGGTGCTGAGTTACCAGTT-3′	57	390	ENSRNOG00000004649
MMP9	5′-CCACCGAGCTATCCACTCAT-3′ 5′-GTCCGGTTTCAGCATGTTTT-3′	56.1	159	ENSRNOG00000017539
NF-κB	5′-CCGAGATAATGACAGCGTGT-3′ 5′-CCTTGGGAACGATATGATGG-3′	58.4	217	ENSRNOG00000023258
TNF-α	5′-ACAAGGCTGCCCCGACTAT-3′ 5′-CTCCTGGTATGAAGTCCGAAATC-3′	60	67	[41]
VEGF	5′-GTTCAGAGCGGAGAAAGCATT-3′ 5′-CTTGCAACGCGAGTCTGTGT-3′	60	80	[42]
MMP2	5′-GCAACAAGTATGAGAGCTGC-3′ 5′-CGGTCATCATCGTAGTTGGT-3′	57	85	ENSRNOG00000016695

**Table 3:** Hematological parameters.

Markers	Normal Control	Arthritic Control	Piroxicam	AM-Metha	AM-Hexa	AM-Etha
Hemoglobin (g/dL)	13.52 ± 1.357	9.142 ± 0.521 ^d^	13.50 ± 0.411 ^a^	13.13 ± 0.096 ^a^	13.99 ± 0.625 ^a^	13.05 ± 0.884 ^a^
RBCs (10^6^/µL)	7.143 ± 0.662	5.417 ± 0.789 ^e^	7.188 ± 0.895 ^b^	7.750 ± 0.684 ^a^	7.503 ± 0.545 ^b^	7.625 ± 1.072 ^a^
WBCs (10^3^/µL)	9.723 ± 0.416	17.46 ± 2.350 ^d^	13.77 ± 1.256 ^c^	14.32 ± 2.062 ^b^	14.04 ± 1.192 ^b^	13.28 ± 0.710 ^c^
Platelets (10^3^/µL)	418.7 ± 54.64	711.5 ± 42.70 ^d^	555.7 ± 27.93 ^a^	550.2 ± 33.58 ^a^	637.0 ± 32.48 ^c^	547.2 ± 37.49 ^a^

Here, ^a^ *p* < 0.001, ^b^ *p* < 0.01 and ^c^ *p* < 0.05 are compared to the arthritic group, whereas ^d^ *p* < 0.001 and ^e^ *p* < 0.01 are compared to the normal control group.

**Table 4:** Identified components of methanolic extract of *A. modesta* from mass chromatograms.

Identified Compounds	Molecular Formula	Retention Time	Molecular Weight (g/mol)	Total (%Age)
2-Propanone, 1-cyclopentyl-	C_8_H_14_O	8.480	126	2.294
5-Hydroxymethylfurfural	C_6_H_6_O_3_	11.502	126	1.582
Phenol,2,4-bis(1,1-dimethylethyl)-	C_14_H_22_O	15.533	206	2.230
Hexadecanoic acid, methyl ester	C_17_H_34_O_2_	20.115	270	2.949
Estra-1,3,5(10)-trien-17β-ol	C_18_H_24_O	20.467	256	7.127
3-(2,6,6-Trimethyl-cyclohex-1-enyl)-propionic acid, methyl ester	C_13_H_22_O_2_	20.876	210	1.943
9,12-Octadecadienoic acid (Z, Z)-, methyl ester	C_19_H_34_O_2_	21.757	294	2.212
Methyl stearate	C_19_H_38_O_2_	21.818	298	3.853
6-Octadecenoic acid, methyl ester, (Z)-	C_19_H_36_O_2_	22.029	296	1.875
9,12-Octadecadienoic acid (Z, Z)-	C_18_H_32_O_2_	22.111	280	2.663
Kaur-16-ene	C_20_H_32_	22.857	272	2.440
17-Norkaur-15-ene, 13-methyl-, (8β, 13β)-	C_20_H_32_	23.340	272	3.198
Podocarp-7-en-3β-ol, 13β-methyl-13-vinyl-	C_20_H_32_O	23.490	288	1.785
1,6,10,14-Hexadecatetraen-3-ol, 3,7,11,15-tetramethyl-, (E, E)-	C_20_H_34_O	23.603	290	1.672
Naphthalene, decahydro-1,1,4a-trimethyl-6-methylene-5-(3-methyl-2,4-pentadienyl)-, [4aS-(4aα,5α	C_20_H_32_	23.890	272	4.854
Phenol,2,2′-methylenebis [6-(1,1-dimethylethyl)-4-methyl-	C_23_H_32_O_2_	24.651	340	2.562
Androst-5-en-17-ol,4,4-dimethyl-	C_21_H_34_O	24.764	302	5.426
Estra-1,3,5(10)-trien-17-one,3-hydroxy-2-methoxy-	C_19_H_24_O_3_	25.419	300	17.137
Podocarp-13-ene-14-glycolic acid, 7-hydroxy-8,13-dimethyl-3-oxo-, -lactone	C_21_H_30_O_4_	25.600	346	1.911
Bolasterone	C_21_H_32_O_2_	25.804	316	1.954
16-Allopregnen-3β-ol-20-one	C_21_H_32_O_2_	26.241	316	1.496
Campesterol	C_28_H_48_O	30.702	400	2.046
Stigmasterol	C_29_H_48_O	30.980	412	4.189
ϒ-Sitosterol	C_29_H_50_O	31.515	414	3.981
Lupeol	C_30_H_50_O	32.450	426	16.618

**Table 5:** Identified components of n-hexane fraction of *A. modesta* from mass chromatograms.

Identified Compounds	Molecular Formula	Retention Time	Molecular Weight (g/mol)	Total (%Age)
2-Pyrrolidinone, 1-methyl-	C_5_H_9_NO	7.455	99	4.1584
Heptadecane,2,6,10,15-tetramethyl-	C_21_H_44_	15.314	296	1.121
Phenol,2,4-bis(1,1-dimethylethyl)-	C_14_H_22_O	15.533	206	1.760
Eicosane,2-methyl-	C_21_H_44_	17.876	296	1.229
Hexadecanoic acid, methyl ester	C_17_H_34_O_2_	20.122	270	2.358
1,2-Benzenedicarboxylic acid, butyl 8-methylnonyl ester	C_22_H_34_O_4_	20.544	362	1.507
7,10-Octadecadienoic acid, methyl ester	C_19_H_34_O_2_	21.757	294	1.569
10-Octadecenoic acid, methyl ester	C_19_H_36_O_2_	21.810	296	1.201
Ethyleneglycol dipelargonate	C_20_H_38_O_4_	23.257	342	2.256
Phenol,2,2′-methylenebis [6-(1,1-dimethylethyl)-4-methyl-	C_23_H_32_O_2_	23.897	340	1.125
Estra-1,3,5(10)-trien-17-one,3-hydroxy-2-methoxy-	C_19_H_24_O_3_	24.651	300	7.974
Bis(2-ethylhexyl) phthalate	C_24_H_38_O_4_	25.404	390	6.118
ϒ-Sitosterol	C_29_H_50_O	25.623	414	41.430
Methylenebis(2,4,6-triisopropylphenylphosphine)	C_31_H_50_P_2_	31.515	484	2.582

**Table 6:** Identified components of ethyl acetate fraction of *A. modesta* from mass chromatograms.

Identified Compounds	Molecular Formula	Retention Time	Molecular Weight (g/mol)	Total (%Age)
Phenol,2,4, bis(1,1-dimethylethyl)-	C_14_H_22_O	15.533	206	1.760
Hexadecanoic acid, methyl ester	C_17_H_34_O_2_	20.115	270	2.004
n-Hexadecanoic acid	C_16_H_32_O_2_	20.469	256	2.677
8,11-Octadecadienoic acid, methyl ester	C_19_H_34_O_2_	21.757	294	2.453
10-Octadecadienoic acid, methyl ester	C_19_H_36_O_2_	21.810	296	1.574
Oleic acid	C_18_H_34_O_2_	22.149	282	1.279
9-Octadeccenoic acid,12-hydroxy-, methyl ester, [R-(Z)]-	C_19_H_36_O_3_	23.505	312	1.472
1-Naphthalenemethanol, Decahydro-5-(5-hydroxy-3-methyl-3-pentenyl)-1,4a-dimethyl-6-methyle	C_20_H_34_O_2_	23.897	306	1.333
Phenol,2,2′-methylenebis [6-(1,1-dimethylethyl)-4-methyl-	C_23_H_32_O_2_	24.651	340	4.530
Androst-5-en-17-ol,4,4-dimethyl-	C_21_H_34_O	24.764	302	1.644
Hexadecanoic acid,1-(hydroxymethyl)-1,2-ethanediyl ester	C_35_H_68_O_5_	25.269	568	1.008
Estra-1,3,5(10)-trien-17-one,3-hydroxy-2-methoxy-	C_19_H_24_O_3_	25.404	300	4.534
Bis(2-ethylhexyl) phthalate	C_24_H_38_O_4_	25.630	390	65.902
Squalene	C_30_H_5_O	27.635	410	0.911
Stigmasterol	C_29_H_48_O	30.973	412	1.033
sϒ-Sitosterol	C_29_H_50_O	31.515	414	1.918
Lupeol	C_30_H_50_O	32.435	426	3.696

## Data Availability

All the data is contained in the manuscript.

## Abbreviations

PGE2: Prostaglandin E2, MMP2: Matrix metalloproteinase 2, MMP3: Matrix metalloproteinase 3, MMP9: Matrix metalloproteinase 9, NF-κB: Nuclear factor kappa chain enhancer of activated B cells, IL6: Interleukin 6, IL1β: Interleukin 1β, TNFα: Tumor necrosis factor α, VEGF: Vascular endothelial growth factor, GC/MS: Gas Chromatography/Mass spectrometry, ELISA: Enzyme-linked immunosorbent assay.
